# Valvular Nature of Strangulated Hernia

**Published:** 1857-08

**Authors:** 


*On the Valvular Nature of Strangulated Hernia.*—Incarcerated hernia, in Prof. Roser's opinion, essentially depends upon a valvular mechanism. The obstruction of the contents of the intestine in the incarcerated portion arises from the folds of the mucous membrane lying valve-like against each other, and preventing the passage of gas, fluids, &c. Looking at the complete obstruction which takes place in the hernia, one might suppose that the parts concerned are compressed as closely as in an artery when tied. But all observation teaches us that no such pressure is here exerted; for while the venous circulation is only partially arrested, the arterial remains uninterrupted. Were it otherwise, indeed, the intestinal fold would become rapidly gangrenous. The question is why, if there is space enough to allow of the circulation in the part to continue, cannot we by pressure return to the contents of the intestine?

The nature of the obstruction may be shown by a simple experiment. If a noose intestine containing some fluid or air, be brought within a ring about the size of the finger, and then pressure be made upon the apex of the noose so as to force the contents against the compressing body, complete obstruction to their passage will be found to prevail. And yet a catheter may be passed beside the intestine, and by drawing the latter a little to one side, a considerable space will be perceived. If pressure be made in front of the encircling ring, the contents of the intestine are forced back; but if we press at the end of the noose, the portion that lies next to the ring is forced against the latter, and the canal is closed. If we open the noose on its convex side, and fill it with water, we may observe the valvular disproportion of the intestinal folds, which resemble the valves of the aorta when acting under water.

Deferring to another occasion the exposition of this theory of the taxis deducible from these views, Prof. Roser now points out the support they give to the operation for hernia, without opening the sac—a procedure he regards one of the greatest improvements in surgery since the days of Pare. He believes it has made little progress in Germany and France, as compared with England, owing to the prevalence of a false theory of strangulation of hernia. In respect to the first of these, too exagerated an idea of the constriction that takes place has been entertained, leading to a belief that the mere dilation of the tendinous margins could not suffice for the return of the distended and indurated hernia. The above experiment, which proves the valvular nature of the obstruction, must surely give more confidence in the efficacy of the external incision. We have not space to follow the author in his description of the anatomy of femoral hernia, and which, indeed, essentially resembles that furnished by Cooper.—[British and For. Med.-Chi. Review.

